# How to Identify and Prioritize Psychosocial Factors Impacting Stress Level

**DOI:** 10.1371/journal.pone.0157078

**Published:** 2016-06-15

**Authors:** Mounia N. Hocine, Karim Aït Bouziad, Patrick Légeron, William Dab, Gilbert Saporta

**Affiliations:** 1 Laboratoire Modélisation, Epidémiologie et Surveillance des Risques Sanitaires, Conservatoire national des arts et métiers, 292, rue Saint Martin 75003 Paris, France; 2 *Stimulus*, 28 rue de Mogador, 75009 Paris, France; 3 Centre d’Etude et De Recherche en Informatique et Communications, Conservatoire national des arts et métiers, 292, rue Saint Martin 75003 Paris, France; Radboud University Nijmegen, NETHERLANDS

## Abstract

We develop a methodological approach to identify and prioritize psychosocial factors (stressors) requiring priority action to reduce stress levels. Data analysis was carried out on a random sample of 10 000 French employees who completed, during a routine interview with the occupational physician, a 25-item questionnaire about stress levels, as well as a questionnaire about 58 stressors grouped into 5 latent variables: job control, job context, relationships at work, tasks performed and recognition. Our method combines Importance-Performance Analysis, a valuable approach for prioritizing improvements in the quality of services, with Partial Least Squares-Path modeling, a Structural Equation Modeling approach widely applied in psychosocial research. Findings on our data suggest two areas worthy of attention: one with five stressors on which decision makers should concentrate, and another with five stressors that managers should leave alone when acting to reduce stress levels. We show that IPA is robust when answers to questions are dichotomized, as opposed to the initial 6-point Likert scale. We believe that our approach will be a useful tool for experts and decision-makers in the field of stress management and prevention.

## Introduction

Work-related stress has become an increasingly major occupational health issue, as it has negative effects on both physical and psychological health [1,2]. Although stress is an inevitable part of organizational life, efforts should be made to reduce its duration and intensity. In order to achieve this, psychosocial risk factors (“stressors”) related to a high level of stress need to be well-documented, and the impact of each stressor needs to be measured in several different ways (not one-dimensionally). Empirical frameworks have been particularly successful in generating and collecting data. Two main models are known to be useful for showing the impact of stress on health: the Demand/Control (or Job Strain) Model, and the Effort/Reward Imbalance Model (ERI) [3–7]. However, there has been limited attention in the literature on the quantitative assessment of stressors' impact on stress levels, taking into account the multidimensional aspect and latent variable nature of this type of data.

Here, we propose a methodological approach to help decision makers implement a strategy to prevent work-related stress. In particular, we develop a quantitative risk assessment method to identify a set of stressors requiring priority action to reduce stress levels in the workplace. Also, an appropriate methodological tool needs to consider multiple existing stressors and the correlation structure that might exist between them, and should thus be based on multivariate data analysis.

Simple indicators such as the Cooper index [8,9] have been used to identify occupational stress predictors for academic staff in Canada [10] and South-Africa [11], but none of these provides a hierarchy of stressors according to their impact on stress, nor takes into account the correlation structure between them.

Here, we suggest combining Importance-Performance Analysis (IPA), a valuable graphical method for prioritizing improvements in the quality of services, and Partial Least Squares-Path modeling (PLS-PM), a Structural Equation Modeling (SEM) approach, widely applied in psychosocial research.

The article is structured as follows. First, we first describe the study data. Second, we show the limitations of the Cooper index [8,9], used to rank stressor's effect independently on stress level. In addition, we describe how SEM, and in particular PLS-PM, may be used to rank five blocks of stressors (latent variables) according to their impact on the stress block. Lastly, we show how IPA, based on the PLS-PM results, may help identify stressors which decision-makers should concentrate on.

## Materials and Methods

### Study data

The present work was motivated by the analysis of a large database belonging to *Stimulus*, an independent French consulting firm specialized in wellbeing and occupational health. *Stimulus* has developed and made available for companies an online tool to collect data on *stress* levels and psychosocial risk factors, and in the future to develop a useful preventative intervention strategy.

#### Data collection

Participants filled in, during the yearly compulsory routine visit with the company's occupational physician, two optional anonymous online questionnaires risk factors. More than 80% of the employees accepted to answer them. The first was a set of questions measuring psychological health (including stress levels), and the second a set of questions measuring psychosocial risk factors contributing to stress level changes. *Stimulus* collected such data anonymously from their French clients, mainly in the field of public services, retail and transport. A sample of 10 000 employees was drawn according to a simple random sampling scheme from hundred thousands of individual's data, collected between 2011 and 2012.

#### Stress measurement tool

A Psychological Stress Measure (S1 Table) [12–14] was used to assess current stress levels. Respondents were asked to rate their perceived state using an 8-point Likert scale ("not at all" to "greatly"). A stress scale was then defined by taking the sum of responses to the 25 questions, thus varying between 25 and 200. This measurement tool was used in the context of evaluating and preventing work-related stress. It has previously been found to have acceptable psychometric properties [13,14].

These 25 items define a block of items associated with a latent variable we name: “*stress*”.

#### Stressors measurement tool

In order to measure psychosocial risk factors at work, *Stimulus* has developed a 58-item questionnaire (S2 Table). This tool is based on an integrative view of various models of stress and psychosocial risk [3–7]. Participants give their degree of agreement with each of the 58 items on a 6-points Likert scale, ranging from 0 (strongly disagree) to 5 (strongly agree).

The 58 items are grouped into five blocks, associated with the following latent variables:

- work context
- job control
- relationships at work
- type of performed tasks
- recognition.

### The Cooper index: limitations and flaws

A method developed by Cooper & Clarke, involving a quantitative risk assessment approach to prioritize psychosocial risks in the workplace [9], is currently often used. To identify stressors related to high stress level, they suggest calculating a “risk level indicator” to measure the impact of a given stressor on stress:

Risk=Exposure(E)×Consequences(C) (1)

Here, E is the mean value of the stressor (exposure) and C (consequences) the proportion of variance of stress level explained by the given stressor (r²) obtained from a simple linear regression.

However, the relevance of this indicator is questionable, despite its ease of use. The “risk level indicator” (1) represents neither a statistical risk measure, nor an impact measure. Indeed, the r² is obtained from a simple linear regression between stress (the outcome) and a given stressor, where the impact of the other stressors is ignored. In short, this would imply that acting on a given stressor to reduce stress levels does not impact the other stressors, which is not entirely reasonable.

**Fig 1:**
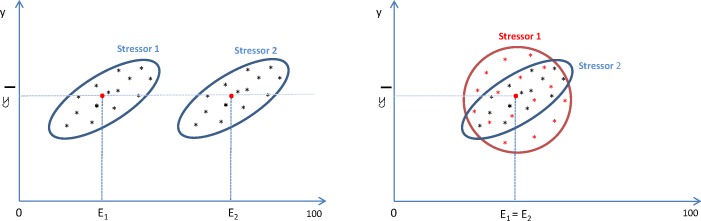
Illustration of variation in stress level y for 2 different stressors x_1_ and x_2._

We illustrate this (Fig 1) with two examples of variation of stress level (y) following exposure to a given stressor (x).

In the left-hand panel, we show the case of two stressors x_1_ and x_2_ with the same variability, as well as the same correlation with y, whereas the average exposure for x_2_ is higher than for x_1_ (E_2_ > E_1_). It follows that x_1_ and x_2_ have the same risk, since increasing (or decreasing) x_1_ or x_2_ equally would have the same influence on y. However, according to Cooper’s index, x_2_ should have a higher risk than x_1_, which is not true. In the right-hand panel, we show the case of two stressors x_1_ and x_2_ with the same average exposure level (E_1_ = E_2_), where variability and correlation with y are different (x_1_ is more correlated with y than x_2_). Therefore, x_2_ has a higher risk than x_1_ because y, given x_2_, has more variability than y, given x_1_. However, according to Cooper’s index, x_1_ should have a higher risk than x_2_, which is not true here.

The mean value of a stressor may be considered as a performance index. However, it is not related to any effect on stress. The slope of the regression line would be a better indicator than the correlation; however, a simple regression is not pertinent here. In the next section, we show that structural equation modeling can provide an adequate solution.

### Structural equation modeling: the PLS-PM approach

**Fig 2:**
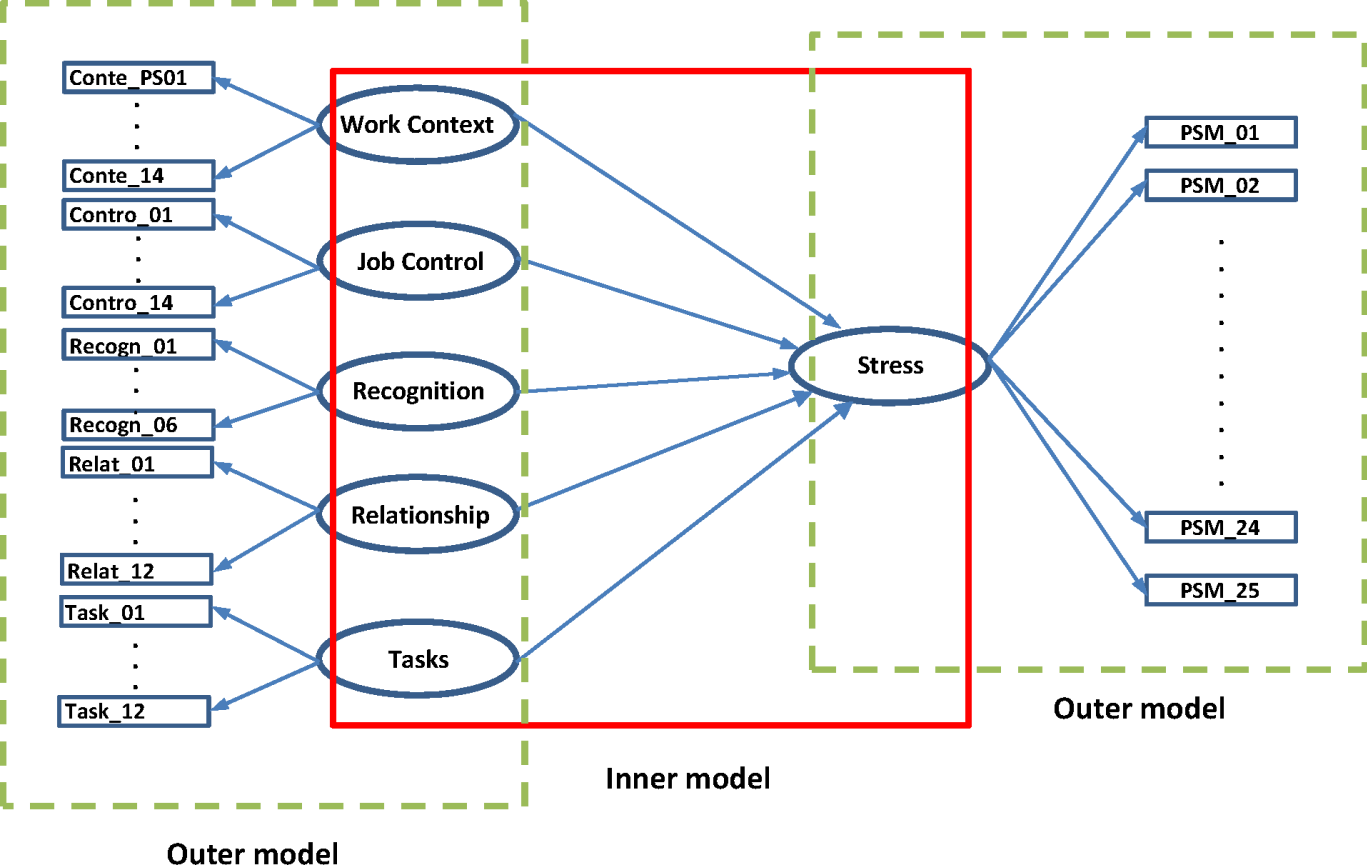
Conceptual model for stressors and work-related stress.

To obtain insight into data on stressors and stress level, correlation analyses were conducted using structural equation modeling (SEM), which has the advantage of providing global measures of fit for latent (subjective or unobserved) variable models [15,16]. The SEM approach is based on conceptual models, and defined by two sets of equations: the inner (or structural) model, and the outer (or measurement) model. The structural model specifies relationships between latent constructs (block of stressors), whereas the measurement model specifies relationships between a latent construct and its manifest variables (stressors), as illustrated in Fig 2. Structural equation models help to assess whether or not hypothesized relationships between variables are valid for the study population, as it enables the specified relationships to be tested against the observed pattern of correlation between variables.

In fact, we hypothesize in the underlying conceptual model, developed with experts at *Stimulus*, that all stressor blocks are closely related (i.e., correlated), and that changes in one would be negatively associated with stress level.

To predict the impact of the 5 job stressors blocks (exogenous constructs) on the stress block (endogenous construct), we used PLS-PM over other SEM approaches, for two main reasons. First, it allows the development of a system of weights via several indices, when the blocks of stressors are strongly correlated, which was the case with our data. Second, PLS-PM is a better choice when the Gaussian distribution assumption is not valid, which is the case for stressors (6-point Likert scale). PLS-PM uses an iterative algorithm; after convergence, latent variable scores are obtained for each observation, and structural coefficients are estimated using multiple regression.

This approach appears to be a useful tool for psychosocial risk management policy for the workplace, and comes with two advantages. First, it allows us to build a relevant scale for stress level using the 25 items in the PSM25 questionnaire, rather than using an equally-weighted scale. Second, it gives a ranking of the five blocks of stressors according to their predictive impact on stress level, using path coefficient values.

However, in order to directly prioritize stressors impacting stress level, we considered constructing a system of levers to identify stressors which decision makers should maintain or act on in priority, to reduce stress level. To achieve this, we suggest using importance-performance analysis (IPA) [17], where weights and path coefficients estimated in the PLS model are used to calculate item importance.

### Importance-performance analysis

IPA is an easily understandable graphical tool presented as a grid divided into four quadrants. The horizontal axis shows the item’s performance, and the vertical axis its importance. The four quadrants are as follows. A is the top-left quadrant (“Concentrate Management Here”), B the top-right (“Keep up the Good Work”), C bottom-left (“Low Priority”) and D bottom-right (“Possible Overkill”).

Of most interest are items in quadrants A and B, as these are relatively “more important” than those in quadrants C and D. Therefore, an item with a lower performance and a higher importance falls into quadrant A, indicating that decision makers should devote further resources to this particular attribute, so as to improve its performance.

An item's performance is defined as its observed mean score over the 10 000 individual responses to this item. Its importance is calculated with the formula (2) in which the importance of a given item is the product of the absolute value of the outer weight and the path coefficient of the latent variable in which the item belongs, obtained from the PLS-PM:

$$\text{Importance}\;({\text{k}}^{\text{th}}\;\text{item})=100\times |\text{Outer weight}({\text{k}}^{\text{th}}\;\text{item of}\;{\text{j}}^{\text{th}}\;\text{block})|\times \text{Path coefficient}({\text{j}}^{\text{th}}\;\text{block},\;\text{stress})$$ (2)

### Ethics statement

The study was approved by the French Data Protection Authority “La Commission Nationale de l’Informatique et des Libertés” (CNIL, #1839949 v 0).

## Results

### PLS-PM results

To evaluate the homogeneity (or unidimensionality) of the six blocks, eigenvalues of the correlation matrix between manifest variables (stressors) belonging to the same block were calculated [18]. Table 1 gives the values of the first eigenvalue corresponding to each block, as well as Cronbach's α. Since the first eigenvalue is dominant and Cronbach's α is high; we may conclude as to the unidimensionality of each block, which can thus be summarized as a single latent variable. The PLS-PM approach, together with the two measurement tools, were validated in the four following steps.

**Table 1:** First eigenvalue for each latent variable and Cronbach's α (N = 10 000).

Latent variables	# items	1^st^ eigenvalue	Cronbach's α
Stress (PSM25)	25	10.92	0.94
Work context	14	5.55	0.88
Job control	14	3.99	0.80
Relationships	12	4.97	0.87
Tasks	12	2.86	0.62
Recognition	6	3.15	0.81

First, correlations between the five blocks of stressors (*work context*, *job control*, *relationships*, *tasks and recognition*), given in Table 2, show that they were mutually positively and strongly related. For this reason mainly, PLS-PM is an appropriate SEM approach to deal with collinearity issues. Indeed, the structural model should be tested for potential collinearity that might bias the results of the underlying multiple regression analysis.

**Table 2:** Correlations between latent variables (N = 10 000).

	Context	Control	Recognition	Relationship	Tasks	Stress
Context	1.00					
Control	0.78	1.00				
Recognition	0.72	0.63	1.00			
Relationship	0.69	0.67	0.60	1.00		
Tasks	0.61	0.72	0.54	0.53	1.00	
Stress	-0.52	-0.63	-0.43	-0.51	-0.52	1.00

Second, the measurement model allowed us to confirm the validity of the six latent constructs. As for the total stress and total stressors, the outer weights were statistically significant, indicating the relevance of the latent variables. Thus, the measurement model quality is satisfactory. Furthermore, normalized outer weights of items belonging to the stress block could potentially be used to define a more relevant stress scale, using the PSM25 questionnaire. This is a more relevant scale than that with all 25 items equally weighted as described above in the Stress measurement paragraph.

**Fig 3:**
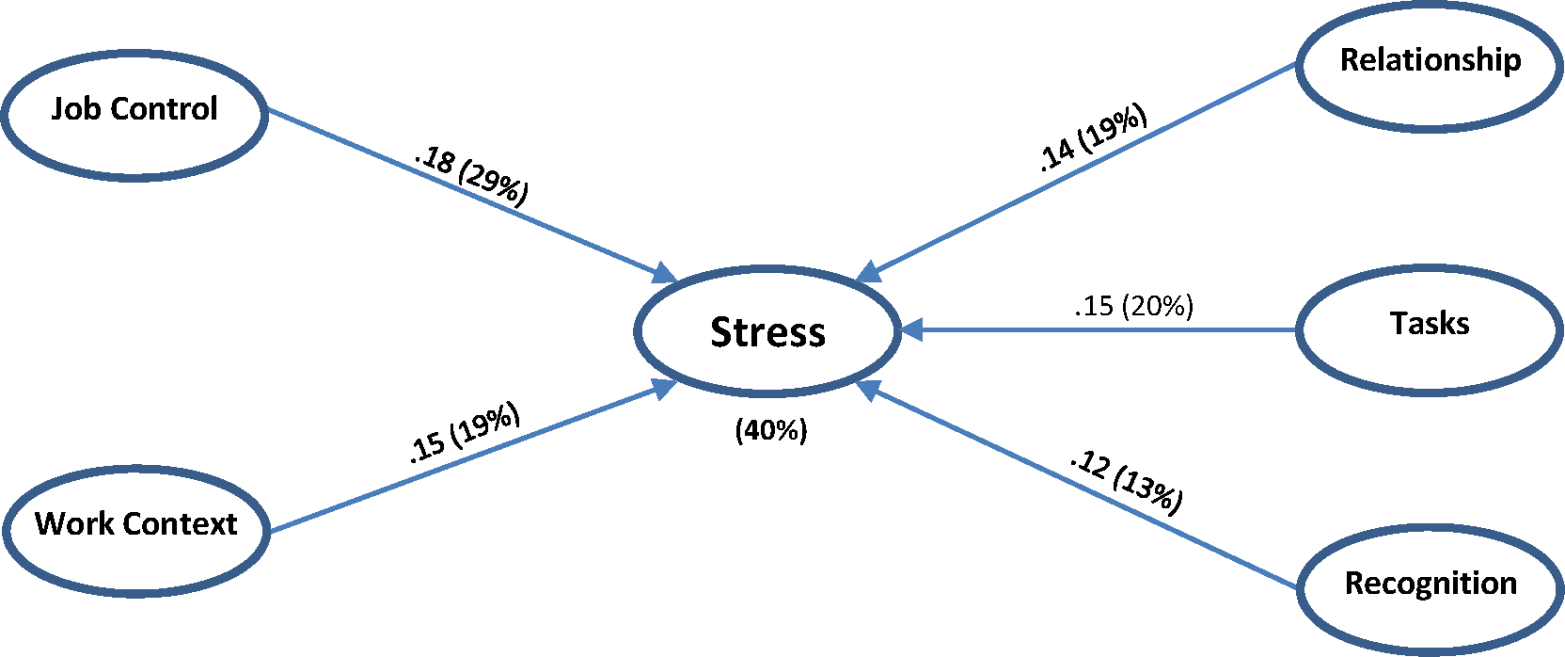
Path coefficients and contributions of each block of stressors to the stress block: β (R²).

Third, the structural model allowed us to evaluate the strength and significance of the path coefficients for the relationships (structural paths) hypothesized between the constructs. The path coefficient values (Fig 3), indicate similar, negative impact of each of the five stressor constructs on the stress construct.

Finally, the assessment of the model’s quality shows the good ability of the model to predict the endogenous construct “stress” (R² = 0.40), indicating that the five blocks of stressors explain 40% of the variance of the stress construct (Fig 3).

We performed PLS-PM as described above, using a 10 000 employees dataset. A randomly drawn subsample of 5 000 employees was used to develop the models, and the remaining 5 000 employees' data served for the validation step. Analyses were performed using the PLS-PM package from the XLSTAT software (https://www.xlstat.com/en/).

### IPA results

**Fig 4:**
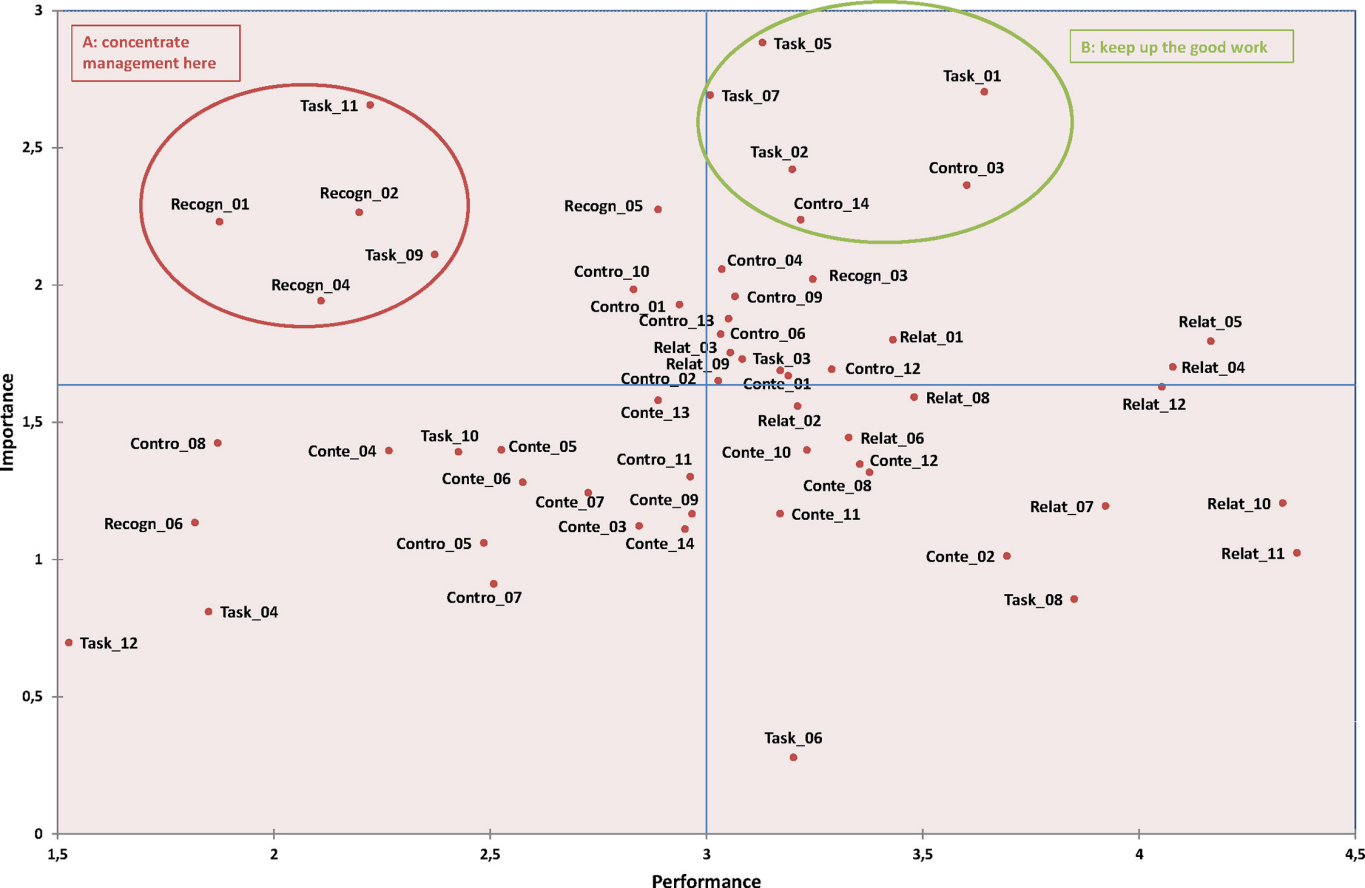
IPA grid for the 58 stressors coded on a 6-point Likert scale.

The IPA plot of importance according to performance values for each of 58 stressors is shown in Fig 4 and S4 Table. Sets of stressors in each of the quadrants A and B were identified, and detailed below.

In terms of the most important items, for the most part the five following items were identified in quadrant A, where improvement in performance is the most pressing, and upon which management should concentrate its efforts:

1. Task_11: « I have to work fast in a short timeframe »
2. Recon_01: « My promotion prospects are weak »
3. Recon_02: « My company offers me interesting career opportunities »
4. Task_09: « I work in a noisy and hectic atmosphere »
5. Recon_04 **: «** I am rewarded when I reach my goals »

Of the most important items, for the most part the following six items were identified in quadrant B, where efforts should be maintained:

1. Task_05: « I frequently see the work pile up without being able to eliminate the backlog »
2. Task_07: « My work gives me many opportunities to perform interesting tasks »
3. Task_01: « My work has meaning to me »
4. Task_02: « My job involves monotonous and repetitive tasks »
5. Contro_03: « I can achieve professional life—personal life balance »
6. Contro_14: « I'm undergoing or I expect to undergo an undesirable change that might affect my career »

These results from the analyzed data suggest that, for organizational strategies to prevent and manage stress, decision-makers should act in priority on the level of the 5 “stressors to improve", and should maintain the level of the 6 “stressors to maintain”, because otherwise the stress level will increase.

### Robustness analysis

IPA’s robustness against changes in the scale used to categorize the answers to the 58 items related to stressors, was tested as follows. Instead of using the 6-point Likert scale for individuals’ answers to each of the 58 items, we *dichotomized* the answers as follows:

- Negative responses: 0 (strongly disagree), 1 (disagree) and 2 (slightly disagree) were coded 0
- Positive responses: 3 (slightly agree), 4 (agree) and 5 (strongly agree) were coded 1

Results (not shown here) demonstrate a similar distribution of the items in the four quadrants. In particular, the items to improve (from quadrant A) and the items to maintain (from quadrant B) were similar. This indicates the robustness of the IPA results.

## Discussion

For the last twenty years, work-related stress has been recognized as a major factor in employee health and company performance. It is now widely recognized that besides biological, chemical and physical agents, the psychosocial working conditions are important determinants of employee health. Many studies have documented the effect of adverse psychosocial work factors on the incidence and prevalence of health problems [6,19]. However, merely providing a list of stressors is not sufficient to define relevant preventive measures. In this context, it is crucial to offer a statistical approach able to identify stressors requiring priority action to reduce stress level, that takes into account the complexity of the relationship between work-related stress and different stressors.

This work aimed to develop a new statistical approach to identify a set of stressors, among many known possibilities, in order to prioritize preventive actions, using two complementary powerful statistical methods: PLS-PM and IPA. To our knowledge, this is the first time these two approaches have been used together to answer a single question. The use of this strategy provides additional insights to understanding the relationship between different stressors and stress level. Our results show that this approach could provide a more relevant diagnosis of stress predictors than Cooper’s index, or any other tool based on univariate statistical analysis.

### Strengths

Although several models are available to identify risk factors impacting stress level, none are based on a solid theoretical base. These models were developed using empirical field studies. However, it is very important to be able to take into account interactions between the various risk factors for stress, identified through epidemiological studies. The PLS-PM approach allows us to predict stress levels using five strongly correlated blocks built from 58 stressors, and to understand concepts that are difficult to formalize. Using path coefficients, this approach allows us to prioritize the five stressor's latent variable constructs based on their ability to predict stress's latent variable construct.

The IPA approach allows the direct identification of stressors requiring priority attention for managerial action to reduce workplace stress levels, using an indirect computation of item importance coming from the PLS-PM results. Based on a plot of item importance in relation to measured performance, IPA provides a useful and easily understandable graphical guide as to how the quadrants differ from one another. As a result, it allows decision-makers to identify areas in which they must reallocate resources [20] in future organizational strategies to prevent and manage stress. Furthermore, the rather similar distribution of items on the IPA plot observed using dichotomized responses as opposed to the initial 6-point Likert-scale indicates the robustness of the IPA results.

The use of data from *Stimulus* provided a two-fold advantage in this study. First, the high quality of data collection, thanks to the large set of study questionnaires filled out during routine visits with company occupational physicians. Second, being able to use such a large dataset (10 000 employees) meant powerful statistical results could be obtained.

### Limitations

As several studies suggest that women suffer more stress than men [21–23], we performed stratified analyses, separately for men (49.6% of the employees) and women (50.4%), and found similar division over the 4 quadrants in the IPA analysis. This means that there is no need to adjust for gender, which would require more complex computations [24]. Other characteristics, either at individual level or at company level, were not considered in our analyses. In fact, *Stimulus* database involved data from hundreds of companies, but with many characteristics, such as profession or type of company, considered confidential.

### Future research

As correlation does not imply causality, a causal analysis should also be performed to determine the stressors on which to act in order to reduce psychosocial disorders associated with high stress level. Bühlmann proposes an analysis based on causal graphs [25] which can be used to supplement the previous approach in finding stressors on which to act in priority to reduce stress levels. This causal model could be validated using longitudinal data collected after putting in place a strategy for intervention against work-related stress prevention.

## Conclusions

We have proposed using a multivariate statistical approach based on IPA combined with PLS-PM. The results from applying this approach to data from *Stimulus* suggest two areas worthy of decision maker attention to reduce stress level. Results also show the robustness of IPA when the answer to each item is dichotomized, compared with the initial 6-point Likert scale.

Our approach could be a useful tool in evaluating the impact of organizational and environmental factors on individual’s stress levels. However, it can used to study any other psychological health outcome or concept (performance, fatigue, anxiety, etc.).

## Supporting Information

S1 Table Psychological Stress Measure PSM25. (DOCX)

S2 Table Psychosocial factor measurements. (DOCX)

S3 Table Cross-loading values and bootstrap intervals. (DOCX)

S4 Table IPA grid: description of the items belonging to each quadrant. (DOCX)

## References

[1] Lee JS, Joo EJ, Choi KS (2013) Perceived Stress and Self‐esteem Mediate the Effects of Work‐related Stress on Depression. Stress and Health 29: 75–81. 10.1002/smi.2428 22610597

[2] Danna K, Griffin RW (1999) Health and well-being in the workplace: A review and synthesis of the literature. Journal of management 25: 357–384.

[3] Siegrist J, Peter R (1994) Job stressors and coping characteristics in work-related disease: issues of validity. Work & Stress 8: 130–140.

[4] Folkman S, Lazarus RS, Dunkel-Schetter C, DeLongis A, Gruen RJ (1986) Dynamics of a stressful encounter: cognitive appraisal, coping, and encounter outcomes. Journal of personality and social psychology 50: 992 3712234 10.1037//0022-3514.50.5.992

[5] Karasek R, Brisson C, Kawakami N, Houtman I, Bongers P, Amick B. (1998) The Job Content Questionnaire (JCQ): an instrument for internationally comparative assessments of psychosocial job characteristics. Journal of occupational health psychology 3: 322 9805280 10.1037//1076-8998.3.4.322

[6] Cooper CL, Marshall J (1976) Occupational sources of stress: A review of the literature relating to coronary heart disease and mental ill health. Journal of occupational psychology 49: 11–28.

[7] Nieuwenhuijsen K, Bruinvels D, Frings-Dresen M (2010) Psychosocial work environment and stress-related disorders, a systematic review. Occupational Medicine 60: 277–286. 10.1093/occmed/kqq081 20511268

[8] Biron C, Ivers H, Brun J-P, Cooper CL (2006) Risk assessment of occupational stress: Extensions of the Clarke and Cooper approach. Health, Risk & Society 8: 417–429.

[9] Clarke SG, Cooper CL (2000) The risk management of occupational stress. Health, Risk & Society 2: 173–187.

[10] Biron C, Brun J-P, Ivers H (2008) Extent and sources of occupational stress in university staff. Work: A Journal of Prevention, Assessment and Rehabilitation 30: 511–522. 18725713

[11] Barkhuizen N, Rothmann S (2008) Occupational stress of academic staff in South African higher education institutions. South African Journal of Psychology 38: 321–336.

[12] Tessier R, Lemyre L, Fillion L (1990) Mesure du stress psychologique (MSP): manuel d’utilisation Editions Behaviora Inc, Brossard, QC.

[13] Fillion L, Tessier R, Tawadros É, Mouton C (1989) Stress et immunité: Étude de validité d'une mesure de stress psychologique (MSP). Canadian Psychology/Psychologie canadienne 30: 30.

[14] Lemyre L, Tessier R (2003) Measuring psychological stress. Concept, model, and measurement instrument in primary care research. Canadian Family Physician 49: 1159 14526870 PMC2214290

[15] Esposito Vinzi V, Russolillo G (2013) Partial least squares algorithms and methods. Wiley Interdisciplinary Reviews: Computational Statistics 5: 1–19.

[16] Esposito Vinzi V, Chin WW, Henseler J, Wang H (2010) Handbook of partial least squares: Concepts, methods and applications.

[17] Martilla JA, James JC (1977) Importance-performance analysis. The journal of marketing: 77–79.

[18] Sijtsma K (2009) On the use, the misuse, and the very limited usefulness of Cronbach’s alpha. Psychometrika 74: 107–120. 20037639 10.1007/s11336-008-9101-0 PMC2792363

[19] Niedhammer I, Chastang J-F, Levy D, David S, Degioanni S, Theorell T. (2008) Study of the validity of a job-exposure matrix for psychosocial work factors: results from the national French SUMER survey. International archives of occupational and environmental health 82: 87–97. 10.1007/s00420-008-0311-7 18327603

[20] Matzler K, Bailom F, Hinterhuber HH, Renzl B, Pichler J (2004) The asymmetric relationship between attribute-level performance and overall customer satisfaction: a reconsideration of the importance–performance analysis. Industrial Marketing Management 33: 271–277.

[21] Matud MP (2004) Gender differences in stress and coping styles. Personality and individual differences 37: 1401–1415.

[22] Aneshensel CS, Pearlin LI (1987) Structural contexts of sex differences in stress.

[23] Baruch GK, Biener L, Barnett RC (1987) Women and gender in research on work and family stress. American Psychologist 42: 130 3578991 10.1037//0003-066x.42.2.130

[24] Chin WW (1998) The partial least squares approach to structural equation modeling. Modern methods for business research 295: 295–336.

[25] Bühlmann P (2013) Causal statistical inference in high dimensions. Mathematical Methods of Operations Research 77: 357–370.

