# Supplementary material for: How to Identify and Prioritize Psychosocial Factors Impacting Stress Level
Source: PLoS One. 2016 Jun 15;11(6):e0157078. doi: 10.1371/journal.pone.0157078 (PMC4909202; doi:10.1371/journal.pone.0157078)
Supplement: S1 Table — (DOCX) [file pone.0157078.s001.docx]

| **PSM 25 items : description FR - ENG** |
| --- |
| Je suis tendu(e) ou crispé(e) - I feel tense or stressed |
| Je me sens la gorge serrée ou j'ai la bouche sèche - I feel that I have a lump in my throat or a dry mouth |
| Je me sens pressé(e) par le temps, coincé(e) par le temps, je manque de temps - I feel rushed; I do not seem to have enough time |
| J'ai tendance à sauter des repas ou à oublier de manger - I tend to skip meals or forget to eat |
| Je ressasse les mêmes idées, rumine, j'ai les mêmes pensées à répétition, la tête pleine - I rehash the same ideas, ruminate, have the same thoughts repeatedly, lots going on in my head |
| Je me sens seul(e), isolé(e), incompris(e) - I feel lonely, isolated, misunderstood |
| J'ai des douleurs physiques: mal au dos, mal à la tête, mal dans la nuque, mal au ventre - I have physical aches and pains: sore back, headache, stiff neck, stomach ache |
| Je suis préoccupé(e), tourmenté(e) ou tracassé(e) - I feel preoccupied, tormented, or  worried |
| J'ai des variations de température corporelle subites (très froid ou très chaud) - I have sudden changes in body temperature (very cold or very hot) |
| J'oublie des rendez-vous, des objets ou des choses à faire - I forget appointments, objects or things to do |
| Je pleure facilement - I cry easily |
| Je suis fatigué(e) - I feel tired |
| J'ai les mâchoires serrées - I have a clenched jaw |
| Je suis calme - I feel calm |
| Je pousse de longs soupirs ou je reprends tout à coup ma respiration - I have long sighs or I take sudden breaths |
| J'ai la diarrhée ou des crampes intestinales ou je suis constipé(e) - I have diarrhea or intestinal cramps or I am constipated |
| Je suis anxieux(se), inquièt(e) ou angoissé(e) - I am anxious, worried or distressed |
| Je sursaute facilement - I’m easily startled |
| Je mets plus d'une demi-heure à m'endormir - I take more than half an hour to fall asleep |
| Je suis confus(e), je n'ai pas les idées claires, je manque d'attention et de concentration - I feel confused; my thoughts are muddled; I lack concentration; I cannot focus |
| J'ai les traits tirés ou les yeux cernés - I look tired or have sunken eyes |
| Je sens beaucoup de pression sur les épaules - I feel a lot of weight on my shoulders |
| Je suis fébrile, j'ai toujours envie de bouger, je ne tiens pas en place - I'm restless, I always need to move, I cannot stand still |
| Je contrôle mal mes réactions, mes humurs, mes gestes - I have difficulty controlling my  reactions, emotions, moods, or gestures |
| Je suis stressé(e) - I feel stressed |
