# Supplementary material for: How to Identify and Prioritize Psychosocial Factors Impacting Stress Level
Source: PLoS One. 2016 Jun 15;11(6):e0157078. doi: 10.1371/journal.pone.0157078 (PMC4909202; doi:10.1371/journal.pone.0157078)
Supplement: S3 Table — (DOCX) [file pone.0157078.s003.docx]

| Latent variable | Manifest variables | Outer weight | Bootstrap 95% CI |
| --- | --- | --- | --- |
| Context | Conte_ 01 | 0.116 | (0.109 , 0.121) |
|  | Conte_ 02 | 0.069 | (0.065 , 0.075) |
|  | Conte_ 03 | 0.077 | (0.070 , 0.083) |
|  | Conte_ 04 | 0.096 | (0.091 , 0.101) |
|  | Conte_ 05 | 0.096 | (0.091 , 0.100) |
|  | Conte_ 06 | 0.088 | (0.082 , 0.092) |
|  | Conte_ 07 | 0.085 | (0.081 , 0.090) |
|  | Conte_ 08 | 0.090 | (0.084 , 0.095) |
|  | Conte_ 09 | 0.080 | (0.076 , 0.084) |
|  | Conte_ 10 | 0.096 | (0.089 , 0.100) |
|  | Conte_ 11 | 0.080 | (0.075 , 0.085) |
|  | Conte_ 12 | 0.092 | (0.088 , 0.098) |
|  | Conte_ 13 | 0.108 | (0.104 , 0.113) |
|  | Conte_ 14 | 0.076 | (0.071 , 0.081) |
| Control | Contro_01 | -0.110 | (-0.115 , -0.104) |
|  | Contro_02 | -0.094 | (-0.099 , -0.088) |
|  | Contro_03 | -0.134 | (-0.141 , -0.127) |
|  | Contro_04 | -0.117 | (-0.122 , -0.112) |
|  | Contro_05 | -0.060 | (-0.070 , -0.054) |
|  | Contro_06 | -0.100 | (-0.105 , -0.094) |
|  | Contro_07 | -0.052 | (-0.058 , -0.045) |
|  | Contro_08 | -0.081 | (-0.088 , -0.074) |
|  | Contro_09 | -0.111 | (-0.119 , -0.105) |
|  | Contro_10 | -0.113 | (-0.118 , -0.107) |
|  | Contro_11 | -0.074 | (-0.080 , -0.066) |
|  | Contro_12 | -0.096 | (-0.101 , -0.091) |
|  | Contro_13 | -0.107 | (-0.114 , -0.100) |
|  | Contro_14 | -0.127 | (-0.134 , -0.119) |
| Recognition | Recogn_01 | -0.186 | (-0.195 , -0.178) |
|  | Recogn_02 | -0.189 | (-0.198 , -0.179) |
|  | Recogn_03 | -0.168 | (-0.178 , -0.159) |
|  | Recogn_04 | -0.162 | (-0.169 , -0.155) |
|  | Recogn_05 | -0.190 | (-0.197 , -0.183) |
|  | Recogn_06 | -0.094 | (-0.107 , -0.084) |
| Relationship | Relat_ 01 | 0.126 | (0.119 , 0.135) |
|  | Relat_ 02 | 0.109 | (0.100 , 0.116) |
|  | Relat_ 03 | 0.127 | (0.118 , 0.136) |
|  | Relat_ 04 | 0.119 | (0.112 , 0.126) |
|  | Relat_ 05 | 0.126 | (0.120 , 0.131) |
|  | Relat_ 06 | 0.101 | (0.094 , 0.107) |
|  | Relat_ 07 | 0.084 | (0.078 , 0.089) |
|  | Relat_ 08 | 0.111 | (0.106 , 0.117) |
|  | Relat_ 09 | 0.121 | (0.115 , 0.127) |
|  | Relat_ 10 | 0.084 | (0.079 , 0.091) |
|  | Relat_ 11 | 0.072 | (0.066 , 0.078) |
|  | Relat_ 12 | 0.114 | (0.107 , 0.122) |
| Tasks | Task_ 01 | -0.186 | (-0.198 , -0.175) |
|  | Task_ 02 | -0.167 | (-0.177 , -0.156) |
|  | Task_ 03 | -0.115 | (-0.132 , -0.095) |
|  | Task_ 04 | -0.056 | (-0.066 , -0.043) |
|  | Task_ 05 | -0.199 | (-0.212 , -0.186) |
|  | Task_ 06 | -0.019 | (-0.032 , -0.009) |
|  | Task_ 07 | -0.186 | (-0.194 , -0.174) |
|  | Task_ 08 | -0.059 | (-0.068 , -0.050) |
|  | Task_ 09 | -0.146 | (-0.155 , -0.133) |
|  | Task_ 10 | -0.096 | (-0.111 , -0.082) |
|  | Task_ 11 | -0.183 | (-0.196 , -0.169) |
|  | Task_ 12 | -0.048 | (-0.065 , -0.036) |
