# Supplementary material for: How to Identify and Prioritize Psychosocial Factors Impacting Stress Level
Source: PLoS One. 2016 Jun 15;11(6):e0157078. doi: 10.1371/journal.pone.0157078 (PMC4909202; doi:10.1371/journal.pone.0157078)
Supplement: S4 Table — (DOCX) [file pone.0157078.s004.docx]

| **Quadrant** | **Item code** | **Items descripton FR - ENG** |
| --- | --- | --- |
| A | Contro_01 | Au travail, j'ai la possibilité de développer mes habiletés personnelles- At work, I have the opportunity to develop my personal skills |
|  | Contro_10 | Les décisions qui ont un impact sur moi sont prises sans que je sois consulté- The decisions that have an impact on me are taken without me being consulted |
|  | Task_ 09 | Je travaille dans une atmosphère bruyante et agitée- I work in a noisy and hectic environment |
|  | Task_ 11 | Je dois travailler très vite étant donné le peu de temps dont je dispose- I have to work fast in a short time-frame |
|  | Recogn_01 | Mes perspectives de promotion sont faibles- My promotion prospects are weak |
|  | Recogn_02 | Les perspectives de carrière que m'offre mon entreprise sont intéressantes- My company offers me interesting career opportunities |
|  | Recogn_04 | Je suis récompensé lorsque j'atteins mes objectifs- I am rewarded when I reach my goals |
|  | Recogn_05 | Au travail, mes efforts sont suffisamment appréciés- At work, my efforts are appreciated fully |
| B | Contro_02 | Je dispose de moyens techniques et matériels suffisants pour faire correctement mon travail- I have sufficient technical and material resources to properly do my job |
|  | Contro_03 | J'arrive à mener de front ma vie professionnelle et ma vie personnelle- I can achieve professional life - personal life balance |
|  | Contro_04 | On m'a fixé des objectifs de travail que je trouve difficiles à atteindre- I was set work goals that I find hard to attain |
|  | Contro_06 | Mes supérieurs sont ouverts à mes idées et suggestions- My superiors are open to my ideas and suggestions |
|  | Contro_09 | Mon travail empiète sur le temps que j'aimerais consacrer à mes activités familiales/personnelles- My work encroaches on the time I would otherwise spend with my family / personal activities |
|  | Contro_12 | Mon travail m'empêche de développer mes connaissances et compétences- My work prevents me from developing knowledge and skills |
|  | Contro_13 | Des événements imprévus m'empêchent souvent de mener mon travail à bien- Unexpected events often prevent me from completing my work |
|  | Contro_14 | Je suis en train de vivre ou je m'attends à vivre un changement indésirable susceptible d'affecter ma carrière- I'm undergoing or I expect to undergo an undesirable change that might affect my career |
|  | Conte_ 01 | J'ai le sentiment que mon service est défavorisé par rapport à d'autres- I feel that my service is unappreciated compared to others |
|  | Task_ 01 | Mon travail a du sens pour moi- My work has meaning to me |
|  | Task_ 02 | Mon travail consiste en des tâches monotones et répétitives- My job involves monotonous and repetitive tasks |
|  | Task_ 03 | Mon métier exige des efforts ou des tâches physiquement pénibles- My job requires physically demanding actions and tasks |
|  | Task_ 05 | Je vois fréquemment le travail s'amonceler sans pouvoir résorber le retard- I frequently see the work pile up without being able to clear the backlog |
|  | Task_ 07 | Mon travail me donne de nombreuses occasions d'effectuer des tâches intéressantes- My work gives me many opportunities to perform interesting tasks |
|  | Relat_ 01 | Mes relations avec mon supérieur sont harmonieuses- My relationship with my supervisor is harmonious |
|  | Relat_ 03 | Il existe des frictions et de la colère entre collègues- There is tension and anger between colleagues |
|  | Relat_ 04 | Je suis en conflit avec mes chefs- I am in conflict with my superiors |
|  | Relat_ 05 | Je suis régulièrement soumis(e) à un harcèlement personnel sous forme de paroles ou de comportements malveillants- I am regularly subjected to personal harassment in the form of words or malicious behavior |
|  | Relat_ 09 | Mes supérieurs m'aident et me soutiennent quand je rencontre des difficultés- My superiors help and support me when I encounter difficulties |
|  | Recogn_03 | Mon responsable se rend compte de mon investissement dans le travail- My manager is aware of my dedication to the job |
| C | Conte_ 02 | Je sais clairement ce que l'on attend de moi au travail- I clearly know what is expected of me at work |
|  | Conte_ 08 | Je manque de consignes claires sur la manière de travailler- I lack clear instructions on how to do my job |
|  | Conte_ 10 | Je suis tiraillé(e) entre des personnes ayant des attentes différentes par rapport à mon travail- I am torn between people with different expectations for my work |
|  | Conte_ 11 | A mon travail, j'ai suffisamment d'occasions de questionner les responsables- In my work, I have enough opportunity to question my supervisors |
|  | Conte_ 12 | Je reçois souvent des demandes contradictoires (opposées) de la part des autres- I often receive conflicting requests from others |
|  | Task_ 06 | Je dois constamment continuer à me former pour rester performant- I constantly have to undergo training sessions to remain effective |
|  | Task_ 08 | Ce que je fais dans mon travail est nécessaire à la bonne marche de l'entreprise- What I do in my work is necessary for my company to function well |
|  | Relat_ 02 | Si le travail devient difficile, mes collègues me viennent en aide- If work becomes difficult, my colleagues come to my aid |
|  | Relat_ 06 | Mes collègues m'apportent l'aide et le soutien qu'il me faut- My colleagues give me the help and support that I need |
|  | Relat_ 07 | Mes relations avec mes collègues sont satisfaisantes- My relationship with my colleagues is satisfactory |
|  | Relat_ 08 | Je peux parler à mon supérieur hiérarchique de quelque chose qui m'aurait attristé(e) ou contrarié(e) au travail- I can speak to my supervisor about anything that has upset or saddened me at work |
|  | Relat_ 10 | Mon supérieur hiérarchique a des comportements irrespectueux ou rudes envers moi- My supervisor is rude or disrespectful towards me |
|  | Relat_ 11 | Mes collègues ont des comportements irrespectueux ou rudes envers moi- My colleagues are rude or disrespectful towards me |
|  | Relat_ 12 | J'ai récemment été témoin de comportements de harcèlement ou d'intimidation sur mon lieu de travail- I have recently witnessed harassment or bullying at my workplace |
| D | Contro_05 | Je peux prévoir dès le début de la journée la manière selon laquelle elle va se dérouler- I can foresee from the beginning of the day the way in which it will unfold |
|  | Contro_07 | On m'impose la plupart du temps des procédures strictes de travail- My job often requires strict work procedures |
|  | Contro_08 | Il ne m'est pas possible de prévoir ce que sera mon métier dans deux ans- It is not possible for me to predict what my job will be in two years |
|  | Contro_11 | J'ai la liberté de décider comment je fais mon travail- I have the freedom to decide how I do my job |
|  | Conte_ 03 | Lorsqu'il y a des changements au travail, je sais clairement comment ils seront appliqués dans la pratique- When there are changes in the workplace, I know clearly how they will be applied in practice |
|  | Conte_ 04 | Mon entreprise est juste et équitable avec ses employés- My company is just and fair with its employees |
|  | Conte_ 05 | J'ai confiance dans la capacité du management à s'occuper de l'avenir de l'entreprise- I have confidence in management's ability to look after the company's future |
|  | Conte_ 06 | La communication au sein de mon entreprise est satisfaisante- Communication within my company is satisfactory |
|  | Conte_ 07 | Les managers de mon entreprise sont encouragés à veiller au bien-être de leurs collaborateurs- My company's managers are encouraged to ensure the well-being of their employees |
|  | Conte_ 09 | Mon responsable traite les problèmes dès qu'ils apparaissent- My manager deals with problems as they arise |
|  | Conte_ 13 | Mon entreprise ne se soucie pas du bien-être de ses salariés- My company does not care about the wellbeing of its employees |
|  | Conte_ 14 | J'ai suffisamment d'occasions de questionner les responsables au sujet des changements au travail- I have sufficient opportunity to question my supervisors about changes at the workplace |
|  | Task_ 04 | Dans mon travail, faire une erreur pourrait avoir des conséquences graves- In my work, making a mistake could have serious consequences |
|  | Task_ 10 | Je dois traiter énormément d'informations complexes et nombreuses- I have to deal a lot with complex and high quantities of information |
|  | Task_ 12 | Le métier que j'exerce nécessite de s'adapter sans cesse à des choses nouvelles- My job requires me to constantly adapt to new things |
|  | Recogn_06 | Mon niveau de rémunération est trop faible par rapport à mes résultats et réalisations- My salary is too low with respect to my performance and achievements |
